# Parallel Gene Expression Differences between Low and High Latitude Populations of *Drosophila melanogaster* and *D*. *simulans*

**DOI:** 10.1371/journal.pgen.1005184

**Published:** 2015-05-07

**Authors:** Li Zhao, Janneke Wit, Nicolas Svetec, David J. Begun

**Affiliations:** 1 Department of Evolution and Ecology, University of California Davis, Davis, California, United States of America; 2 Department of Bioscience, Section of Integrative Ecology and Evolution, Aarhus University, Aarhus C, Denmark; University of Southern California, United States

## Abstract

Gene expression variation within species is relatively common, however, the role of natural selection in the maintenance of this variation is poorly understood. Here we investigate low and high latitude populations of *Drosophila melanogaster* and its sister species, *D*. *simulans*, to determine whether the two species show similar patterns of population differentiation, consistent with a role for spatially varying selection in maintaining gene expression variation. We compared at two temperatures the whole male transcriptome of *D*. *melanogaster* and *D*. *simulans* sampled from Panama City (Panama) and Maine (USA). We observed a significant excess of genes exhibiting differential expression in both species, consistent with parallel adaptation to heterogeneous environments. Moreover, the majority of genes showing parallel expression differentiation showed the same direction of differential expression in the two species and the magnitudes of expression differences between high and low latitude populations were correlated across species, further bolstering the conclusion that parallelism for expression phenotypes results from spatially varying selection. However, the species also exhibited important differences in expression phenotypes. For example, the genomic extent of genotype × environment interaction was much more common in *D*. *melanogaster*. Highly differentiated SNPs between low and high latitudes were enriched in the 3’ UTRs and CDS of the geographically differently expressed genes in both species, consistent with an important role for cis-acting variants in driving local adaptation for expression-related phenotypes.

## Author Summary

While gene expression variation in natural populations is common, the population genetic processes responsible for the maintenance of this variation remain obscure. Here we study geographic differences in gene expression in recently established low and high latitude populations of two closely related species of *Drosophila*. We observe substantial parallelism in expression differences and expression plasticity between populations, which supports the idea that spatially varying selection correlated with latitude contributes to the maintenance of gene expression variation in these species. Comparison of inter-population sequence differentiation and expression differentiation suggests that cis-acting variants play a role in geographic expression differentiation.

## Introduction

Parallel adaptive evolution in natural populations has been of longstanding interest to evolutionary biologists because its prevalence speaks to the repeatability of adaptive trajectories or mechanisms and in doing so, informs our understanding of the general “rules” of divergence under selection. Indeed, convergent phenotypic evolution is now typically considered *prima facie* evidence of adaptation [1]. Much empirical support for parallel adaptive evolution at both the phenotypic and molecular level comes from documentation of evolutionary changes on the relatively long timescale of interspecific divergence [1–5]. Alternatively, relatively few studies provide insight into the question of whether recent, potentially transient adaptive processes occurring within species, perhaps associated with spatially varying selection, are repeated in different species [6–10].

In *Drosophila melanogaster*, extensive phenotypic, cytological, and genomic investigations of latitudinal differentiation have revealed widespread signals of local adaptation in North America and Australia [11–15] following colonization out of an African ancestral range [11,16–18]. Less well understood is whether *D*. *simulans*, the sister species of *D*. *melanogaster*, also shows latitudinal differentiation, and if so, whether there is substantial overlap in the targets of spatially varying selection in the two species. *D*. *simulans* is hypothesized to have had an ancestral origin in Madagascar [19] and like *D*. *melanogaster*, only recently colonized Eurasia, North America and Australia [11]. The two species are broadly sympatric human commensals, and can often be simultaneously collected in the field. Like *D*. *melanogaster*, *D*. *simulans* exhibits high levels of nucleotide diversity and low levels of linkage disequilibrium, consistent with large population sizes [20,21]. Unlike *D*. *melanogaster*, which is polymorphic for a number of clinally varying paracentric chromosome inversions [22,23], *D*. *simulans* segregates no common chromosome inversions [24]. Despite this major cytological difference between the two species, their recent shared ancestry and general population genetic similarities suggest the possibility that their evolutionary response to recent colonization of novel habitats might be similar in magnitude and perhaps even overlap substantially in the genetic details. Interestingly, however, the relatively few studies of latitudinal variation in *D*. *simulans* provide little support for this expectation. For example, studies of protein electrophoretic variation and life-history traits indicate that *D*. *simulans* exhibits less geographic differentiation than does *D*. *melanogaster* [12,25,26]. Arthur et al. 2008 [27] revealed scant evidence for latitudinal differentiation in heat or cold tolerance in Australian populations of *D*. *simulans*, despite the fact that these traits are strongly correlated with latitude in *D*. *melanogaster* [28]. However, latitudinal clines in wing size in both *D*. *simulans* and *D*. *melanogaster* on multiple continents support the hypothesis that larger body size is favored at higher latitude [27,29–32]. A relatively unbiased and large-scale approach for generating hypotheses regarding the extent and biological basis of parallel latitudinal adaptation in the two species is characterization of gene expression variation.

Here we take this approach to ask whether there is substantial overlap of gene expression divergence associated with high and low latitude American population samples in both species. We also ask whether the geographic differentiation in the influence of temperature variation on gene expression is shared between the two species, which would support the idea that variation in gene expression plasticity is also shaped by spatially varying selection in the two species.

## Results

### Transcriptome Overview of Low and High Latitude *D*. *melanogaster* and *D*. *simulans*

To identify genes that are differentially expressed between low and high latitude populations, we characterized population variation in whole male transcriptomes of *D*. *melanogaster* and *D*. *simulans* at 21°C and 29°C using sympatric isofemale strains established from flies collected in Maine and Panama. We created three biological replicates for each species × population × temperature combination for a total of 24 libraries. Sequences were generated by 90 bp paired-end Illumina Hiseq. After filtering, we obtained 347.7 million paired reads for *D*. *melanogaster* and 359.4 million paired reads for *D*. *simulans* (S1 Table). Estimates of transcript abundance (Fragments Per Kilobase Of Exon Per Million Fragments Mapped, FPKM) were highly correlated across biological replicates (Pearson’s r correlation ~ 0.99 and 0.98 for *D*. *melanogaster* and *D*. *simulans*, respectively, P <2.2E-16, S1 Fig).

### Differential Gene Expression in High vs. Low Latitude Populations

**Fig 1:**
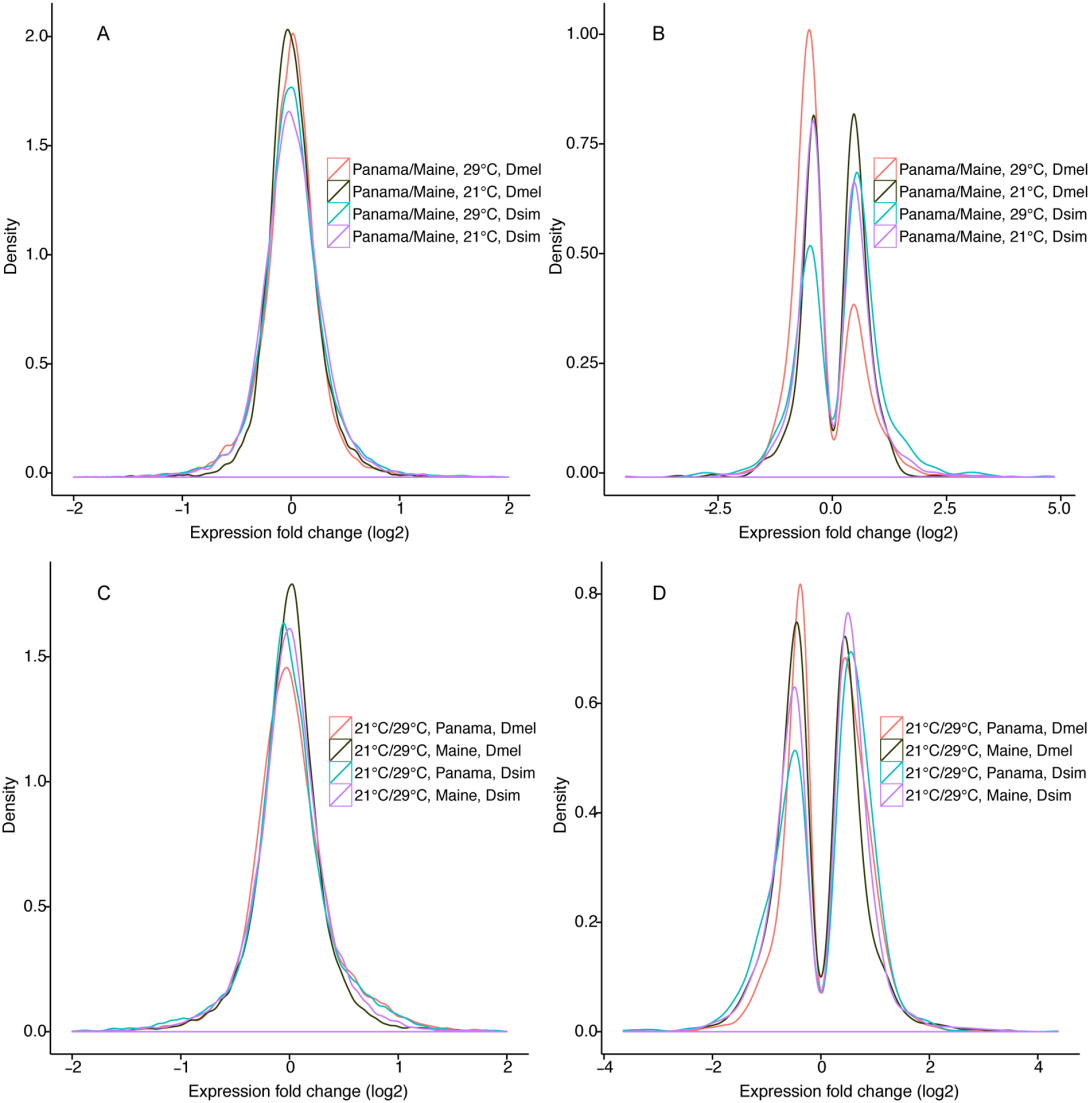
Expression fold changes for each comparison in *D*. *melanogaster* and *D*. *simulans*. A) Fold changes (log2) for Panama vs. Maine population at 21°C and 29°C. B) Fold changes (log2) for differentially expressed genes in Panama vs. Maine population at 21°C and 29°C. C) Fold changes (log2) for 21°C vs. 29°C in each population. D) Fold changes (log2) for genes showing differential expression at 21°C vs. 29°C in each population.

In *D*. *melanogaster*, 759 (5.4%) and 980 (6.9%) genes were significantly differentially expressed between populations (False discovery rate (FDR) <0.05) at 21°C and 29°C, respectively (Table 1, Fig 1A and 1B, S2 Fig, S2 Table). The magnitude of expression differences was significantly greater at 29°C than at 21°C. At 21°C, similar numbers of differentially expressed genes showed higher expression in Maine vs. lower expression in Maine (Fig 1B, dark green line, 396 vs. 363, *χ* ^*2*^ test, P >0.05), while at 29°C most differentially expressed genes showed higher expression in Maine than in Panama (Fig 1B, red line, 681 vs. 299, *χ* ^*2*^ test, P <0.001). Only 193 genes were differentially expressed between populations at both temperatures, supporting the idea that expression differences between these populations are not solely attributable to a similar generalized stress response for Panama at 21°C and Maine and 29°C. Moreover, GO enrichment analysis (below) provided no evidence that stress response pathway genes were enriched among differentially expressed genes.

**Table 1:** Panama vs. Maine differential expression.

Species	Temperature	Mean fold change, all genes	Mean fold change, differentially expressed genes	Differentially expressed genes (FDR 0.05)	Differentially expressed one-to-one orthologs (FDR 0.05)	Differentially expressed male-biased genes	P-value
*D*. *melanogaster*	29°C	14.6%	56%	980 (6.9%)	717 (7.1%)	393 (10.0%)	6.8E-18
	21°C	13.2%	47%	759 (5.4%)	523 (5.2%)	239 (6.1%)	0.0158
*D*. *simulans*	29°C	16.6%	66%	821 (6.1%)	634 (6.3%)	219 (6.2%)	0.4241
	21°C	16.7%	54%	1206 (8.9%)	921 (9.1%)	355 (10.0%)	0.0061

Numbers of expressed genes were 14,006 in *D*. *melanogaster* and 13,464 in *D*. *simulans*; 10,085 expressed genes were one-to-one orthologs between species. Fold change corresponds to mean absolute fold change. Male biased genes exhibited at least 2-fold expression difference between males and females and minimum expression estimate FPKM>2; 3,920 and 3,546 male-biased genes were identified in *D*. *melanogaster* and *D*. *simulans*, respectively. P-values derive from hypergeometric test for overrepresentation of male-biased genes.

We used DAVID [33] and GOTermFinder [34] analysis for functional category enrichment using all differentially expressed genes. At 21°C differentially expressed genes were enriched in response to toxic substance (P = 1.88E-07), response to insecticide (P = 7.92E-07), detection of visible light (P = 0.003), electron carrier activity (P = 0.001), and cytochrome P450s (P = 0.02) (S3 Table). Cytochrome P450 genes were previously inferred to be differentiated between low and high latitude populations in genomic analyses [23,35] and in expression analysis [36,37]. At 29°C differentially expressed genes were significantly enriched in multiple light-stimulus related functional categories, including phototransduction (P = 1.44E-11), detection of visible light (P = 2.66E-09), sensory perception of light stimulus (P = 6.48E-07) and transmission of nerve impulse (P = 0.002, S3 Table). At 29°C, 5 of the top 10 differentially expressed genes (ranked by adjusted P-value) were *trp* and *trp*-interacting *inaF* genes (*inaF-A*, *inaF-B*, *inaF-C*, and *inaF-D*; note that the *inaF* genes may not be independent as they are co-localized in a small genomic region. The fundamental role of TRP proteins in the light-induced calcium channel in *Drosophila* photoreceptors [38] further supports the idea that the visual system is influenced by spatially varying selection. Interestingly, previously published genomic data [15] from the USA provide evidence of two strongly differentiated *trp* non-synonymous SNPs as well as differentiated SNPs in the 5’-flanking region. Focusing on the top 50 differentially expressed protein-coding genes at 21°C and 29°C (S4 Table), eight (C*yp12d1-d*, *Cyp12d1-p*, *dy*, *dsf*, *CG15221*, *inaF-B*, *inaF-D*, and *CG7884*) were differentially expressed at both temperatures; several of the genes showing the greatest expression differences between populations are associated with response to environmental stimulus (S4 Table). Of the 585 expressed annotated transcription factors (TFs), 22 (3.7%) and 39 (6.6%) were differentially expressed between populations (S5 Table) at 21°C and 29°C, respectively. These TFs include *srp*, which is associated with wing size variation in Australian *D*. *melanogaster* populations [39]. Interestingly, 13 of the 22 differentially expressed transcription factors, including *eyeless* (*ey*) and *glass* (*gl*), are associated with eye development and vision. This is potentially noteworthy given the observed high levels of expression differentiation for vision-related genes. Four transcription factors, *pros*, *dsf*, *az2*, and *CG32006* were differentially expressed at both temperatures.

One possible explanation for differences in transcript abundance in whole males from different populations is the presence of population differences in relative size of different organs or tissues, though we are unaware of studies documenting such variation. To address this issue, albeit indirectly, we estimated tau [40,41], an index of tissue specificity, for each gene using FlyAtlas data [42]. We also determined for each expressed gene the tissue that showed the highest expression. If expression differences between populations were attributable primarily to geographic variation in relative sizes of certain organs or tissues, we would expect differentially expressed genes to be enriched with genes showing more tissue expression bias. However, we found no such enrichment for either somatic or testis expression (*χ* ^2^ tests, all P >0.1). Moreover, we found no correlation between tau and fold change for all expressed genes or for significantly differentially expressed genes (Pearson’s R^2^ <0.01). Taken together, these results do not support the idea that high and low latitude population expression differences were driven by geographic differences in relative organ size.

Significantly differentiated genes were not neighbors more often than expected by chance (empirical P >0.1). Additionally, the median physical distance between differentially expressed genes was not significantly different from the expected value assuming such genes were randomly distributed on each chromosome arm (P >0.1, see Methods), which suggests that expression differentiation is primarily a genic rather than a chromosomal region effect. Firmer conclusions on this matter await comprehensive analysis of population genomic and functional datasets.

Previous analysis of the *D*. *melanogaster* transcriptome revealed that roughly 30% of genes show a two-fold or greater difference in expression between the sexes [43,44], primarily as a result of male-biased expression. To determine whether male-biased genes are more likely than other genes to be differentially expressed between populations, we used a two-fold expression difference in modENCODE data from whole adult males vs. females [44] as the cut-off to categorize a gene as male-biased, and then compared this list to the genes expressed in our experiments. We found that a greater proportion of male-biased genes (6.1% at 21°C and 10.0% at 29°C) were differentially expressed between populations compared to other genes (5.2% at 21°C and 5.8% at 29°C; hypergeometric test, both P <0.001, Table 1). This is consistent with previous results that male-biased genes tend to exhibit greater expression variation than do other genes [36,45]. To investigate whether this enrichment is associated with gonadal expression, we estimated the proportion of differentially expressed genes between populations for testis-biased genes (as defined in [41]) and for other genes. This comparison revealed no evidence of increased likelihood of differential expression for testis-biased genes (hypergeometric test, both P >0.1, S6 Table), which suggests that the enrichment observed for male-biased genes is driven by somatically expressed, sexually dimorphic genes [43,46]. In support of this, we found no evidence that male-specific genes [44], which are typically testis-specific in expression, were enriched among the genes showing expression differences between populations (hypergeometric test, both P >0.1, S6 Table).

In *D*. *simulans*, 1206 (8.9%) and 821 (6.1%) genes were differentially expressed between populations at 21°C and 29°C, respectively (FDR <0.05, Fig 1A and 1B, S2 Fig, S2 Table). Thus, compared to *D*. *melanogaster*, *D*. *simulans* shows evidence of substantially more geographic expression differentiation at 21°C and slightly less geographic differentiation at 29°C. As was true for *D*. *melanogaster*, the proportion of genes differentially expressed between populations was significantly different for 21°C vs. 29°C (*χ* ^*2*^ test, P <0.001), though unlike *D*. *melanogaster*, this proportion was higher at 21°C rather than 29°C (Table 1). Similar to *D*. *melanogaster*, magnitudes of expression differences for significantly differentially expressed genes were greater at 29°C (Table 1). Unlike *D*. *melanogaster*, in *D*. *simulans* we observed no difference between temperature treatments with respect to the mean expression difference between populations for all expressed genes (Wilcoxon rank sum test, P = 0.79). At 29°C, slightly more differentially expressed *D*. *simulans* genes showed higher expression in Panama than in Maine (Fig 1B, light blue line, 473 vs. 348, *χ* ^*2*^ test, P = 0.002), while at 21°C, a similar number of genes showed higher vs. lower expression in Maine and Panama (Fig 1B, purple line, 623 vs. 583, *χ* ^*2*^ test, P >0.1), similar to the pattern observed in *D*. *melanogaster*.

In *D*. *simulans* the only significantly over-represented GO terms among differentially expressed genes were structural constituent of cuticle (S3 Table, P = 0.002 at 29°C, P = 0.001 at 21°C) and structural molecule activity (P = 1.77E-04 at 29°C, P = 0.04 at 21°C). Thus, although *D*. *simulans* exhibits somewhat greater geographic differentiation for transcript abundance, the associated genes appear to be less obviously biased across gene functions compared to *D*. *melanogaster*. To investigate whether the relatively incomplete GO annotations in *D*. *simulans* contributed to the small number of enriched GO terms, we also used the GO terms associated with *D*. *melanogaster* orthologs to perform the enrichment analysis and found the same pattern (S3 Table), suggesting that species difference may reflect a real biological phenomenon. In *D*. *simulans*, 13 and 7 of 197 expressed TFs were differentially expressed at 21°C and 29°C, respectively. Two transcription factors, *GD15807* (orthologous gene of *D*. *melanogaster acj6*) and *GD11523* (orthologous gene of *D*. *melanogaster lms*), were differentially expressed between populations at both temperatures.

To investigate whether male-biased genes in *D*. *simulans* are more likely than other genes to be differentially expressed between populations, we used the same criteria as those used in *D*. *melanogaster* (Methods) to categorize genes as male-biased or not. We found that at 21°C a greater proportion of male-biased genes (10.0%) were differentially expressed between populations compared to other genes (8.6%; hypergeometric test, P = 0.006, Table 1) similar to the pattern observed in *D*. *melanogaster*. However, at 29°C we observed no difference in the proportion of differentially expressed genes for male-biased (6.2%) vs. other genes (6.1%) (hypergeometric test, P >0.1).

### Chromosomal Distribution of Geographically Differentially Expressed Genes

In *D*. *melanogaster*, we observed no over or under-presentation of significantly geographically differentially expressed genes on the *X* chromosome vs. the autosomes (*χ* ^*2*^ test, P >0.1, Table 2). However, considering each chromosome arm separately, *3R* was enriched for geographically differentially expressed genes (*χ* ^*2*^ test, P <0.001, Table 2) at both experimental temperatures. In *D*. *melanogaster* the frequencies of cosmopolitan chromosome inversions are often negatively correlated with latitude [14,47,48]. These inversions appear to have relatively small genomic effects on the magnitude of genomic differentiation between high and low latitude populations [15,49], with the exception of chromosome *3R*, which is substantially more differentiated than the other arms [14,48]. This *3R*-effect appears to be driven in large part by *In(3R)Payne*, which shows steep latitudinal clines [47,48]. To investigate the possible influence of cosmopolitan inversions on gene expression differentiation between populations, we compared the proportion of differentially expressed genes in regions spanned by *In(3R)P*, *In(3R)Mo*, *In(3L)P*, *In(2L)t*, *In(2R)Ns*, and *In(3R)K* relative to autosomal regions not spanned by inversions. For both temperatures, we observed a greater proportion of differentially expressed genes in regions spanned by *In(3R)P* and *In(3R)K* compared to other chromosomal regions (hypergeometric tests, P <0.001, S7 Table), supporting the idea that chromosome inversions contribute to expression differentiation on *3R*. This concordance between genomic differentiation and expression differentiation for *3R* supports the idea that cis-acting regulatory variants play an important role in gene expression differences between populations, which is expected given the importance of such variants for expression variation within populations [41,50]. Unlike *D*. *melanogaster*, there are no common chromosome inversions segregating in *D*. *simulans* [24]. *D*. *simulans* exhibited no heterogeneity across chromosome arms in the proportion of differentially expressed genes at either temperature. However, relative to autosomal genes, *X*-linked differentially expressed genes were slightly underrepresented at 29°C but not at 21°C (Table 2).

**Table 2:** Chromosomal distribution of geographically differentially expressed genes.

Species	Chromosome	Gene number	Panama vs. Maine at 29°C	Panama vs. Maine at 21°C
*D*. *melanogaster*	Total	14006	980	759
	*2L*	2721	160	128
	*2R*	2846	174	136
	*3L*	2633	172	143
	*3R*	3402	294***	216***
	*X*	2169	157	105
	*U* and *4*	235	23	31
*D*. *simulans*	Total	13464	821	1206
	*2L*	2371	159	212
	*2R*	2576	153	234
	*3L*	2450	154	225
	*3R*	3253	203	278
	*X*	1601	70**	130
	*U* and *4*	1213	82	127

P-values were calculated by *χ* ^*2*^ test.

** P<0.01

*** P <0.001

### Parallel Expression Differences between High and Low Latitude Populations in *D*. *melanogaster* and *D*. *simulans*

**Fig 2:**
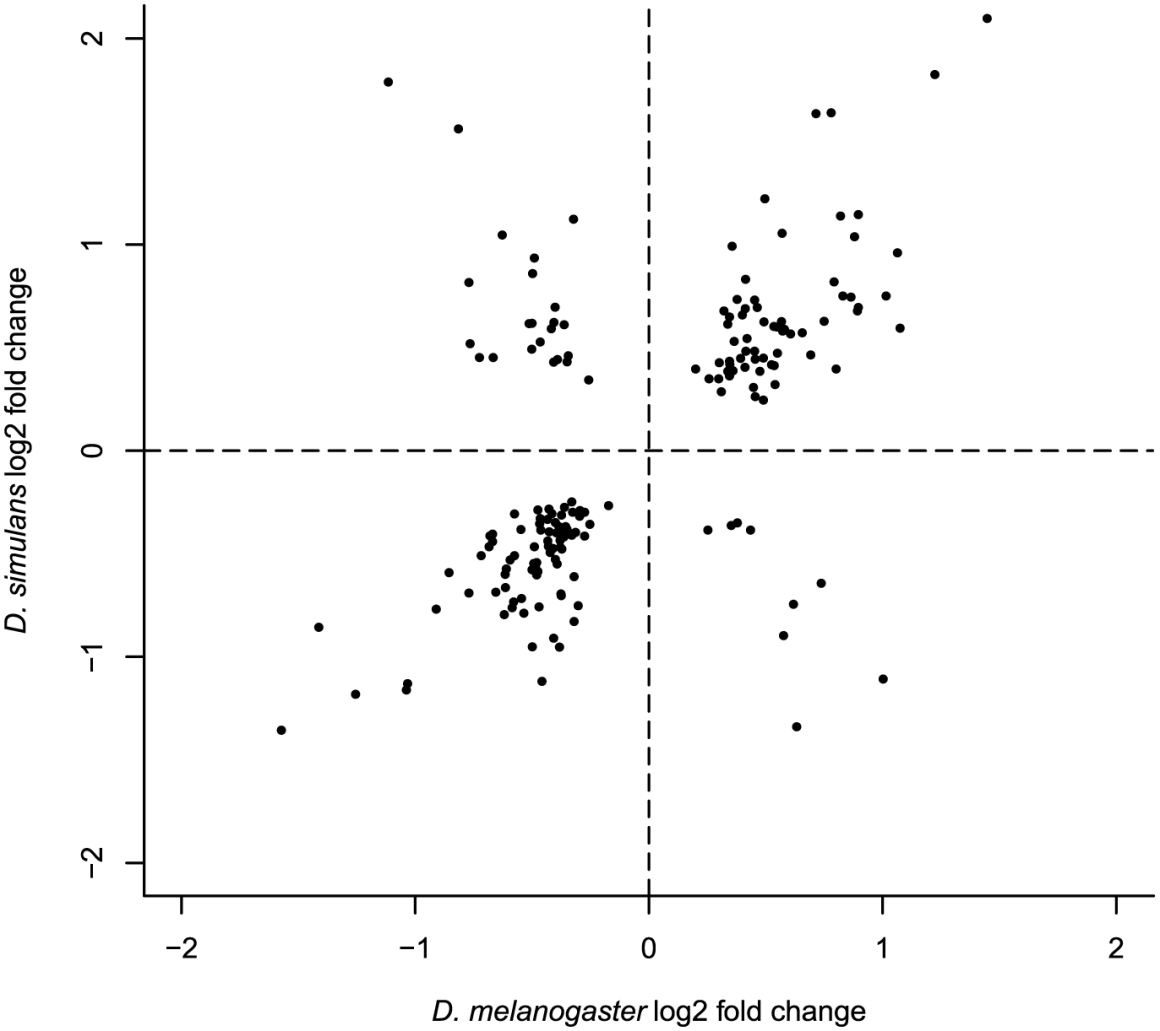
Log2 fold expression change correlation between *D*. *melanogaster* and *D*. *simulans* for genes exhibiting parallel expression differentiation (7 outliers are not presented but included in Pearson’s correlation calculation).

To investigate whether these closely related species exhibit parallel patterns of geographic expression differentiation (possibly associated with the colonization of new habitats), we compared differentially expressed genes in Maine vs. Panama samples from both species, using only one-to-one orthologs that satisfied our minimum expression criteria in all samples (10,085 genes). We observed 106 and 76 genes that were geographically differentially expressed in both species for 21°C and 29°C, respectively (Table 3, S8 Table). This 12.1%-26.5% overlap corresponds to a 2-to-3 fold enrichment, (hypergeometric test P <8.92E-12 for 29°C, P <3.45E-31 for 21°C), which supports the idea that a significant component of geographic differentiation in gene expression is driven by local adaptation occurring in a similar manner in these two species, though it is worth keeping in mind that a majority of differentially expressed genes is not shared between species. The fact that most (82.5%) shared, differentially expressed genes exhibit the same direction of differential expression (both species exhibit higher expression in Panama or both species exhibit higher expression in Maine) (S8 Table, Fig 2), strongly supports the proposition that parallel geographic differentiation reflects parallel adaptation. There were no significantly enriched GO terms associated with these shared differentially expressed genes (FDR >0.1) for either temperature, though the small number of genes provides little power for detecting such enrichments. Five genes, *tim* (a circadian rhythms gene), *Cyp6a19* (a cytochrome P450), *Mur18b* (a chitin binding gene), *CG34461* (a chitin-based cuticle gene), and *CG17752*, showed significant geographic differentiation in gene expression in both species at both temperatures. The T-box transcription factor *Doc1* was geographically differentially expressed in both species, showing higher expression in Maine at 21°C. Three likely transcription factors (based on ortholog gene function), *GD10401* (ortholog of *CG30431*), *GD20175* (ortholog of *gl*), and *GD14098* (ortholog of *CG6765*) showed significant geographic differentiation in both species, all with higher expression level in Maine at 21°C. Genes that showed parallel expression differentiation exhibited no significant heterogeneity across chromosome arms (hypergeometric tests, all P >0.05). Among the genes showing parallel gene expression differences between populations there was no obvious trend for transcript abundance to be greater in either Panama or Maine (*χ* ^*2*^ test, P >0.1). Finally, among all expressed one-to-one orthologs, we observed a highly significant excess of parallel expression differences (5851 parallel genes vs. 4617 opposite direction genes at 29°C, 6591 parallel vs. 3594 opposite genes at 21°C, *χ* ^*2*^ test, both P <0.001), which supports the proposition that parallel selection responses may shape expression differences even for genes that are not significantly differentially expressed in our data.

**Table 3:** Panama vs. Maine parallel gene expression differences in *D*. *melanogaster* and *D*. *simulans*.

Temperature	*D*. *melanogaster* genes	*D*. *simulans* genes	Shared genes	P-value
29°C	626	543	76	8.92E-12
21°C	399	789	106	3.46E-31

10,085 one-to-one orthologs satisfied our minimum expression criteria in all samples. *D*. *melanogaster* and *D*. *simulans* genes refer to geographically differentially expressed genes that have one-to-one orthologs. “Shared genes” correspond to one-to-one orthologs differentially expressed in both species. P-values were generated by hypergeometric test.

To investigate whether parallelism extends to the magnitude of population differences in transcript abundance, we estimated the interspecific correlation in fold change for genes exhibiting parallel significantly different expression. While the biased subset of data included makes it difficult to evaluate the correlation, the observed correlation (Pearson’s R^2^ of log2 fold changes = 0.80, Fig 2, S1 Text) appears to be remarkably high. This suggests (though by no means proves) that the relationship between expression variation and fitness variation may be quite similar for the genes influenced by spatially varying selection in the two species.

### Parallel Differentiation in Gene Expression Plasticity

In *D*. *melanogaster*, 1329 (9.5%) and 2788 (19.9%) genes showed differential expression between 21°C and 29°C for Maine and Panama, respectively (Fig 1C and 1D, S3 Fig, Table 4, S9 Table), while 770 genes showed significant expression plasticity for both populations. The differences in plasticity between populations (9.5% vs. 19.9%) were highly significant (*χ* ^*2*^ test, P <0.001), consistent with a previous microarray analysis of high and low latitude Australia populations [51]. In Maine, genes exhibiting significant temperature plasticity were enriched in structural constituent of chitin-based cuticle (P = 9.36E-4), signal peptides (P = 6.96E-04) and muscle protein (P = 2.86E-06). There was an underrepresentation of *X*-linked (relative to autosomal) genes showing expression plasticity for both populations (hypergeometric test, Panama P = 2.97E-05; Maine P = 6.92E-06; shared genes P = 3.06E-05), consistent with the trend reported for African and European populations [36]. Chromosome *3R* was enriched for genes showing expression plasticity, but only in the Panama sample (hypergeometric test, P = 5.60E-07); this pattern is attributable to the enrichment of differentially expressed genes for inversions *In(3R)K* and *In(3R)P*. Focusing on the top 50 most differently expressed genes for temperature plasticity, 24 were shared between Panama and Maine populations, including the transcription factors *ftz* and *twi*.

**Table 4:** Differential gene expression at 21°C vs. 29°C within populations.

Species	Population	Total gene fold change	Differentially expressed gene fold change	Differentially expressed genes	Differentially expressed genes with one-to-one orthologs
*D*. *melanogaster*	Panama	21.1%	53.1%	2788 (19.9%)	2051 (20.3%)
	Maine	17.1%	54.6%	1329 (9.5%)	954 (9.5%)
*D*. *simulans*	Panama	21.1%	65.1%	2147 (16.0%)	1709 (16.9%)
	Maine	19.2%	59.2%	1488 (11.1%)	1179 (11.7%)

Numbers of expressed genes were 14,006 in *D*. *melanogaster* and 13,464 in *D*. *simulans*; 10,085 expressed genes were one-to-one orthologs between species. Fold change refers to the mean absolute fold change. “Total gene fold change” refers to mean absolute expression change for all expressed genes, “Differentially expressed gene fold change” refers to mean absolute expression change for differentially expressed genes.

In *D*. *simulans* of the 13,464 expressed genes, 1488 (11.1%) and 2147 (16.0%) showed differential expression between temperatures for Maine and Panama, respectively (Fig 1C and 1D, Table 4, S9 Table); 951 genes exhibited expression plasticity for both populations. Thus, as was the case for *D*. *melanogaster*, there appears to be greater expression plasticity for Panama than for Maine (*χ* ^*2*^ test, P <0.001). Similar to the differences between species for geographic differentiation in expression at each experimental temperature, *D*. *simulans* exhibits greater expression plasticity than does *D*. *melanogaster* (*χ* ^*2*^ test, P <0.001) for the Maine samples. However, compared to *D*. *melanogaster*, *D*. *simulans* exhibited a smaller proportion of genes with expression plasticity for Panama (*χ* ^*2*^ test, P <0.001). For *D*. *simulans*, there was no significant difference in plasticity between *X* and autosome, however, chromosome *3R* was enriched for differentially expressed genes in both populations (Panama, hypergeometric test, P = 2.96E-06; Maine, hypergeometric test, P = 0.01), in spite of the fact that unlike *D*. *melanogaster*, the *D*. *simulans* pattern cannot be associated with inversion polymorphism. Focusing on the top 50 most differently expressed genes for temperature plasticity, 6 and 9 genes in Panama and Maine, respectively, were involved in chitin-based cuticle development. Other highly differentially expressed genes in both Panama and Maine populations include the *D*. *simulans* orthologs of *CG7214*, *Ance-3*, *tim*, *Rh5*, *retinin*, *Acyp*, and *boss*, some of which have functions related with visual perception. Note that *Ance-3* and *Acyp* are adjacent, suggesting the possibility of shared regulatory information associated with plasticity.

To compare expression plasticity in the two species, we restricted our analysis to one-to-one orthologs that satisfied our minimum expression criteria in all samples. Plasticity was highly conserved between species. For example, for Maine, 375 of 909 genes (41.25%) showed expression plasticity in both species (S10 Table), while for Panama, 861 of 1995 (42.16%) genes exhibited plasticity for both species (S10 Table). For Maine, all 375 genes that showed plasticity for both species exhibited the same direction of differential expression (*i*.*e*., both species showed higher expression at 21°C or both showed higher expression at 29°C). Of these 375 genes, 207 showed higher expression at 29°C while 168 showed higher expression at 21°C (*χ* ^*2*^ test, P = 0.17). For Panama, among the 861 genes that showed plasticity for both species, 830 (96.4%) exhibited the same direction of differential expression; 282 and 548 of these genes showed greater at 29°C and 21°C, respectively. Thus, in Panama, there were significantly more shared genes that show higher expression at 21°C (*χ* ^*2*^ test, P <0.0001). We observed 229 genes showing temperature plasticity in Maine and Panama for both species; 105 and 117 showed higher expression at 21°C and 29°C, respectively. Only 7 of these 229 genes showed different directions of expression plasticity across species, further supporting the idea that expression plasticity is generally conserved.

To compare geographic differences in expression plasticity in the two species, we identified for each species, the genes showing temperature related expression plasticity in either Maine or Panama (but not both) and then determined whether the same genes tend to show population-specific plasticity in the two species. Remarkably, the geographic differences in plasticity exhibited significant parallelism. There were 1804 (17.9%) and 1298 (12.9%) genes that showed geographic differences in plasticity in *D*. *melanogaster* and *D*. *simulans*, 380 (21.1% and 29.3%) of which were shared between the two species, which represents an at least 1.64 fold enrichment (hypergeometric test, P = 1.42E-27). Thus, there is a component of plasticity variation that is highly conserved and another component showing evidence of recent, parallel local adaptation.

Finally, we compared our *D*. *melanogaster* transcriptome data to previously published expression microarray data on expression plasticity for high and low latitude Australian *D*. *melanogaster* populations [51] reared at 18°C and 30°C. We compared the top 300 differentially expressed genes in Innisfail (Queensland, low latitude) to differentially expressed genes in Panama, and found that 129 genes were shared between the two populations. Similarly, the Cygnet (Tasmania, high latitude) and Maine populations shared 117 genes with temperature plasticity (S11 Table). 112 of 129 (86.8%) and 108 of 117 (92.3%) of the above genes showed the same pattern of increased vs. decreased expression in response to temperature for our data from Maine and Panama compared to Australia populations. This suggests, not surprisingly given the parallel temperature response differentiation observed between species, that high *vs*. low latitude *D*. *melanogaster* populations in the Americas and Australia exhibit similar patterns of gene expression temperature response.

### Genetic Differentiation Associated with Geographic Variation in Transcript Abundance

Given previous work supporting the importance of cis-acting variants on gene expression variation in *D*. *melanogaster* [41,50,52,53], we investigated whether genes exhibiting geographic differences in transcript abundance also tend to harbor differentiated SNPs. To do so, we used our transcriptome data to estimate SNP frequencies in Maine and Panama [54] and then estimated *F*_*ST*_ (see methods). We found that genes differentially expressed at 29°C were enriched for highly differentiated SNPs (0.5% *F*_*ST*_ outlier) in the 3’UTRs, 5’UTR and CDS regions, while there was significant enrichment of outlier *F*_*ST*_ SNPs at 3’UTR and 5’UTR for genes differentially expressed at 21°C (Table 5, S12 Table, S13 Table), consistent with our observation that there is greater geographic differentiation at 29°C than at 21°C (Table 1). To determine whether the magnitude of genetic differentiation was associated with the magnitude of gene expression differentiation, we estimated for all expressed genes and for significantly differentially expressed genes the proportion of SNPs in the 0.5% and 0.25% tail *F*_*ST*_ and found no significant correlation between *F*_*ST*_ and expression differentiation for either gene set (Pearson’s *r* <0.1, ANOVA P >0.1). There are many possible explanations for the lack of such a correlation including various limitations of our data and the possible genetic complexity of regulatory variation and its interaction with fitness across genes.

**Table 5:** Outlier SNPs (0.5% *F_ST_* tail) in differentially expressed genes and enrichment P-values.

Species	Temperature	Gene annotation	Number differentially expressed genes	Number differentially expressed genes with tail SNP for different annotations	Enrichment P-value
*D*. *melanogaster*	21°C	3' UTR	531	41	0.009
	21°C	5' UTR	471	29	0.002
		CDS	634	79	0.080
	29°C	3' UTR	692	79	3.69E-11
		5' UTR	623	32	0.014
		CDS	818	112	0.003
*D*. *simulans*	21°C	3' UTR	611	37	0.050
	21°C	5' UTR	623	22	0.482
		CDS	1091	147	0.003
	29°C	3' UTR	382	23	0.114
	29°C	5' UTR	369	16	0.201
		CDS	703	91	0.044

The comparisons for differential expression were Panama vs. Maine. “Number differentially expressed genes” refers to the number of differentially expressed genes that have annotated UTRs or CDS. P-values were from hypergeometric test.

Because estimates of SNP differentiation from RNA-seq data may be influenced by expression differentiation (depending on linkage disequilibrium between cis-eQTLs and nearby SNPs), we extended the analysis of genetic and expression differentiation using previously published genomic data from Maine and Florida populations of *D*. *melanogaster* [15]. We compared our significantly differentially expressed genes to the genes overlapping the 1% most extreme 1-kb *F*_*ST*_ windows in the genome [15]. We observed highly significant overlap between the two sets of genes at 29°C (P = 3.58E-07) and marginally significant overlap at 21°C (P = 0.001), which is consistent with our SNP-based analysis. These results are consistent with an important role for cis-acting variants in gene expression differences between populations [55,56].

The *D*. *simulans* genome annotation is of substantially lower quality than that of *D*. *melanogaster*, leading to reduced power to detect phenomena such as annotation enrichments. Nevertheless, we wanted to determine if this species also exhibits evidence of genetic differentiation in differentially expressed genes. We found that 3’ UTRs and CDSs of expressed genes differentially expressed at 21°C were significantly enriched with highly differentiated SNPs (Table 5) but only CDS regions were enriched with *F*_*ST*_ outliers at 29°C. Thus, similar to the pattern observed in *D*. *melanogaster*, the temperature associated with a greater number of differentially expressed genes tends to exhibit greater enrichment of SNP outliers.

Focusing on the outlier SNPs in the shared differentially expressed genes (106 at 21°C and 76 at 29°C, with a total of 177 genes), we observed that the corresponding genes were enriched for outlier SNPs to a greater extent than all differentially expressed genes (37 for *D*. *melanogaster*, 46 for *D*. *simulans*, 10 were shared, *χ* ^*2*^ test, P = 0.018). Inspection of outlier SNPs in the shared differentially genes revealed no SNPs at homologous sites in the two species, consistent with relatively low levels of shared polymorphism in these species [57].

To investigate parallel geographic differentiation more broadly, we used data from all 10,085 expressed one-to-one orthologs to identify the SNPs in the top 0.5% and 0.25% of the *F*_*ST*_ distribution and their corresponding genes and gene annotation (UTRs or CDS). We first examined the 6867 one-to-one orthologs associated with UTR SNPs in both species. The 0.25% tail UTR SNP *F*_*ST*_ outliers were associated with 304 and 163 genes in *D*. *melanogaster* and *D*. *simulans*, respectively; the 12 genes shared between species represents a marginally significant (P = 0.057) enrichment. The comparable analysis using the 0.5% tail UTR outliers, yielded 555 and 337 genes in *D*. *melanogaster* and *D*. *simulans*, respectively, of which 42 were shared between species (P = 0.002). We then examined the 9479 orthologous genes with CDS SNPs in both species. We observed an excess of shared genes associated with the top 0.25% CDS SNPs (570 genes in *D*. *melanogaster*, 661 genes in *D*. *simulans*, 62 shared, P = 2.66E-05) and 0.5% (1032 genes in *D*. *melanogaster*, 1136 genes in *D*. *simulans*, 197 shared, P = 1.64E-12). These shared genes are associated with substantially longer CDS than other genes. To address the possibility that the excess of shared genes is simply an artifact of gene size, we created an empirical distribution by sampling gene sets of the same size as the observed data and for which the number of SNPs in each gene matched the number in the observed genes such that the total number of SNPs and distribution of SNPs across genes was exactly the same in the observed data and the sampled gene sets. We repeated this 1000 times in each species to generate an empirical distribution of the number of genes harboring at least one shared 0.25% CDS *F*_*ST*_ SNP. We found that the expected (median) number of shared CDS was 36 CDS, while the observed number of shared CDS was 62 (P <0.001). Of the 2032 and 1766 0.25% outlier SNPs identified in *D*. *melanogaster* and *D*. *simulans*, respectively, only 2 were outliers in both species.

### Splice Junction and Isoform Differentiation

In *D*. *melanogaster* we observed 191 annotated splice junctions in 175 genes that showed significantly different expression in Maine vs. Panama at 21°C. At 29°C we observed differential usage of 732 splice junctions in 546 genes (S14 Table). Thus, as was the case for transcript abundance, it appears that there is more differentiation for alternative splice junction use at 29°C. In *D*. *simulans*, we observed 870 splice junctions (in 743 genes) that differed in abundance between Maine and Panama at 21°C and 432 (in 383 genes) at 29°C (S14 Table). This, too, is consistent with the transcript abundance data, and shows the two species have different degrees of expression differentiation at these two temperatures. We observed 20 and 21 splice junctions showing differential expression in both species at 21°C and 29°C, respectively (S15 Table); 37 of the 41 shared splicing junctions shared the same expression direction (for example both showed higher expression in Maine or Panama) in both species, which supports the idea that they have been influenced by parallel spatially varying selection.

We estimated relative isoform usage across *D*. *melanogaster* samples to formally identify possible instances of alternative transcripts exhibiting geographic differentiation. We found 373 and 414 isoforms differentially expressed at 21°C and 29°C, respectively. For most such genes, significant variation was observed only for one transcript. Furthermore, most of these differentially expressed alternative transcripts affected only the choice of UTR. This is consistent with version 5.55 of the *D*. *melanogaster* annotation, for which only 25.5% (3557 of 13937) genes were associated with multiple protein isoforms (the remaining isoforms differing only in the UTRs).

### Population × Temperature Interaction

**Fig 3:**
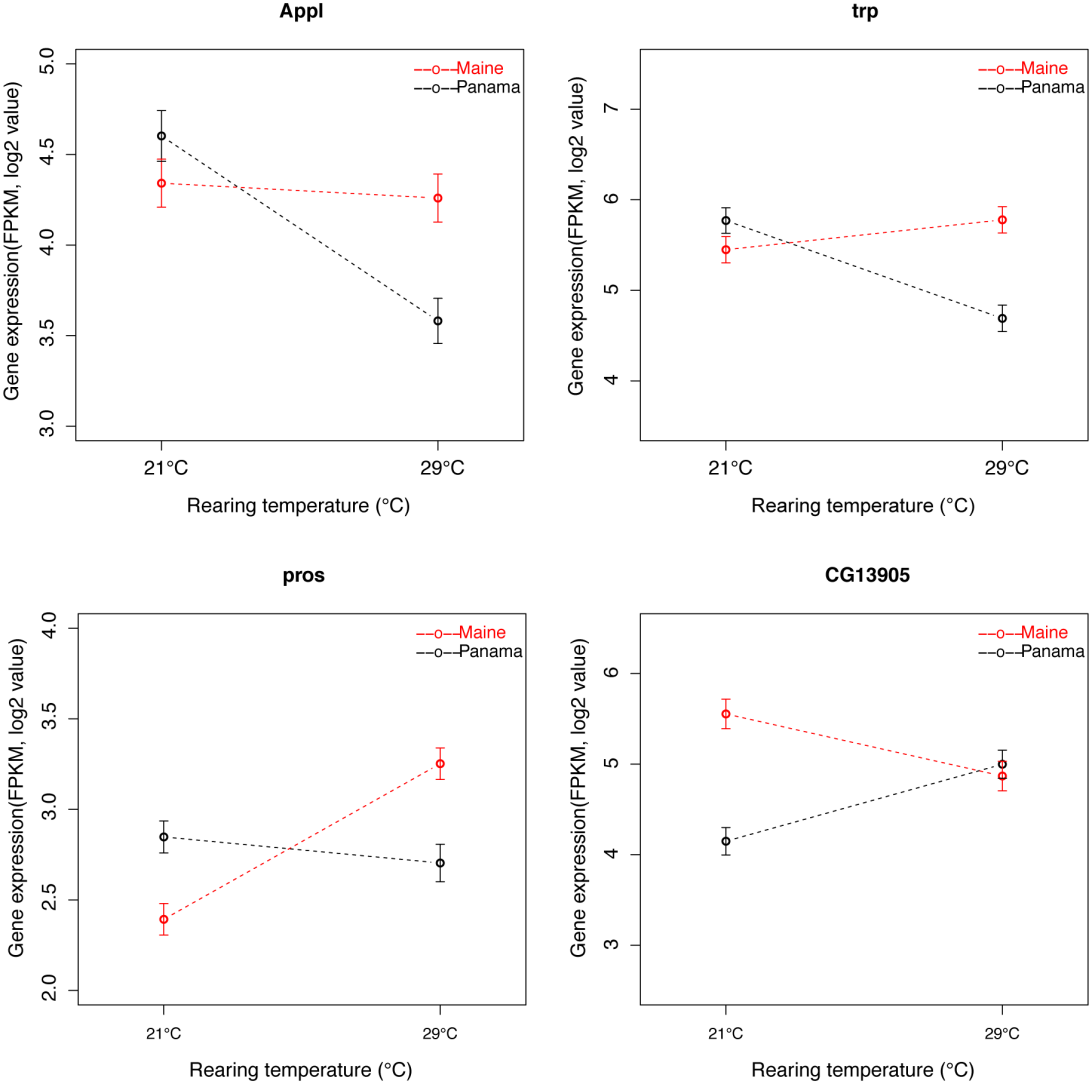
Example of *D*. *melanogaster* genes exhibiting population-by-temperature interaction. *Appl* and *trp* represent typical genes showing greater expression differences at 29°C than 21°C.

Gene-by-environment interactions (GEI) may play a role in the maintenance of genetic variation in the presence of spatially varying selection, even if the optimum phenotype does not vary with geography [58]. While we did not use defined individual genotypes in our experiments, we estimated population × temperature interactions as (Maine at 21°C-Maine at 29-C)-(Panama at 21°C-Panama at 29°C) following Levine et al. [51]; for convenience we refer to this as GEI. At an FDR of 0.05, 264 *D*. *melanogaster* genes showed significant GEI (S16 Table, Fig 3). GO analysis showed that these genes were highly associated with neurological system process, visual perception, sensory perception of light stimulus, and signal transduction (P <0.05, FDR<0.05, S17 Table), consistent with our finding that genes associated with vision show substantial geographic expression differences. Using the same approach in *D*. *simulans*, only 7 genes showed significant GEI (11 genes with FDR increased to 0.1, S16 Table). Thus, it appears that the species are quite different in the extent of GEI across the genome. To further investigate this potential species difference, we summarized gene expression data from both species to identify genes for which the rank order of transcript abundance in the two populations differs across temperatures (and further requiring the log2 of the sum of the absolute difference of the fold difference for the two temperatures to be greater than 0.5; genes satisfying these criteria can be thought of as showing the classic crossing of reaction norms). We observed 754 and 622 such genes in *D*. *melanogaster* and *D*. *simulans*, respectively. Thus, the proportion of genes showing this pattern is only slightly greater in *D*. *melanogaster* (*χ* ^*2*^ test, P = 0.004), though the magnitude of the effect is substantially greater than it is in *D*. *simulans*. To further characterize the difference between species we determined the proportion of the 754 and 622 genes for which the slopes of the lines connecting the expression estimate for each temperature were of different sign (positive vs. negative) in the two populations. In *D*. *melanogaster*, 644 of the 754 genes showed such a pattern while only 268 of 622 genes showed such a pattern in *D*. *simulans*. Focusing on 200 genes exhibiting the greatest population × temperature interaction in each species, 196 *D*. *melanogaster* genes showed the classic crossover between populations at 21°C and 29°C while fewer than 150 *D*. *simulans* genes showed such a pattern. These patterns all support the idea that population × temperature interactions are of greater magnitude in *D*. *melanogaster* than in *D*. *simulans*.

## Discussion

The most important conclusion from the data and analyses reported here is that these two species exhibit significant parallelism with respect to low vs. high latitude gene expression differences. The most parsimonious explanation for the quantitative parallelism is that for a subset of the genome the relationship between transcript abundance and fitness variation across heterogeneous environments is similar in the two species. Given previous population genomic analyses of these species [14,15] and the short timescales of colonization of the Americas for both species [11], selection on standing variation is likely to underlie geographic expression differences for both species. Whether or not the observed expression differentiation is associated with spatially varying selection in the ancestral ranges of the species is an open question. In both species, differentially expressed genes show enrichment for differentiated 3’UTR SNPs. Given the possible role of 3’ UTRs in regulating transcript abundance [59,60] and in light of previous findings of high levels of geographic differentiation in 3’UTRs in whole genome analyses [49], the hypothesis that such cis-acting variation is responsible for some of the parallel gene expression differentiation is worth investigating. While there is little evidence for shared differentiated 3’UTR SNPs in genes exhibiting parallel expression differences in the two species, which is consistent with previous analyses of these two species [57], the more general question of the possible role of parallel non-coding regulatory variation at the level of individual SNPs or regulatory elements in the two species awaits more comprehensive genetic and genomic analyses. It is also worth noting that we observed a substantial number of transcription factors that exhibited expression differences between populations; their role in generating the patterns observed here also remains to be determined.

In addition to the observed interspecific parallelism for transcript abundance, we found substantial parallelism for geographic differences in expression plasticity in the two species. This supports the hypothesis that spatially varying selection influences plasticity, in line with previous results suggesting that selection is the dominant process shaping variation in *Drosophila* [61]. We found for both species, greater expression plasticity for Panama than for Maine populations. Though the classic view is that selection may favor genotypes associated with greater phenotypic plasticity in temperate environments [62], our results do not support that view. If the regulation of expression plasticity often involves trans-acting elements [63,64] then the geographic patterns of expression plasticity observed here may be attributable to trans-acting elements such as transcription factors. Such hypotheses are certainly amenable to testing by further population genomic and functional analysis.

In spite of the evidence for parallelism, there are many differences between species with respect to differentially expressed genes. Moreover, the degree of functional annotation enrichment associated with expression differences is much greater in *D*. *melanogaster* than in *D*. *simulans*. One interpretation of this difference is that selection is distributed across a much greater range of biological functions in *D*. *simulans* than in *D*. *melanogaster*, which would be remarkable given their very recent ancestry and sympatric distribution in recently colonized habitats. Unfortunately, an alternative explanation for this difference is simply that the quality of functional annotation in the two species is very different, though our attempt to circumvent this limitation by using the GO terms associated with *D*. *melanogaster* orthologs for *D*. *simulans* did not show greater enrichment either. A high quality annotation of *D*. *simulans* will be indispensable for incisive analysis of similarities and differences between these species. Nevertheless, we can state with some confidence that the visual system of *D*. *melanogaster* appears to be influenced by strong spatially varying selection, while *D*. *simulans* reveals little evidence of a comparable phenomenon. We do not have any particularly attractive hypotheses to explain this difference. However, we note that one apparently major difference in the visual ecology of these species is that *D*. *melanogaster* requires no light for successful courtship while *D*. *simulans* does [65]. Alternatively, because the visual system has multiple functions including detection of visible and non-visible (UV) light, temperature response (such as TRP-dependent cold/hot response, see review [66]), locomotor [67,68], and circadian behaviors [68] or photoperiodism [69], it is difficult to formulate clearly articulated hypotheses on the possible interspecific differences in ecology that may impinge upon the local adaptation via the visual system in the two species. Cross-talk between pathways for perception of temperature and light [70,71] further complicates the situation. Finally, we cannot rule out a contribution of geographic differences in relative eye size to the observed GO enrichment in *D*. *melanogaster*. In *D*. *simulans*, we found cuticle genes are enriched among the genes showing expression differences between temperatures and between populations, while no such enrichment was observed in *D*. *melanogaster*. A recent genomic analysis [72] showed that cuticle genes in *D*. *simulans* are overrepresented among recently duplicated genes which may have undergone rapid adaptive evolution in *D*. *simulans*. Geographic variation in gene copy number could certainly be related to some of the observations reported here.

Another obvious genomic differences between species was that in *D*. *simulans* a greater proportion of genes showed geographic expression differences at 21°C, while in *D*. *melanogaster* a greater proportion of genes showed expression differences at 29°C. It is difficult to interpret this difference given only the available data, but it would certainly be of great interest to carry out comparable experiments on populations from the ancestral ranges of both species to investigate how the geography of ancestral and recently established populations might illuminate this species difference.

Another noteworthy species difference is the substantially greater population temperature interactions observed in *D*. *melanogaster* than in *D*. *simulans*. A possible explanation for these differences is that optimal *D*. *melanogaster* expression phenotypes are similar in high and low latitude populations experiencing different environments; under such circumstance alleles exhibiting GEI might be favored and exhibit different allele frequencies but the associated genotypes would have similar phenotypes. In this way of thinking, the weaker GEI in *D*. *simulans* is a consequence of the fact that compared to *D*. *melanogaster*, high and low latitude populations more often have different optimal expression phenotypes. Of course, such speculation offers no explanation for why these species might have such different expression/fitness functions in high vs. low latitude environments.

Finally, and in contrast to most previous studies of *D*. *simulans*, our results reveal as much (or more) geographic differentiation in *D*. *simulans* as in *D*. *melanogaster*. Perhaps the conventional wisdom that *D*. *simulans* exhibits less phenotypic clinality than does *D*. *melanogaster* (which is based on relatively few data) needs to be further evaluated [73] ideally in multiple environments. Additional studies, perhaps guided by genomic inferences such as those presented here, might provide evidence of parallel latitudinal differentiation for a great many intermediate phenotypes.

## Materials and Methods

### Sample Preparation

*D*. *melanogaster* and *D*. *simulans* females were collected from Fairfield, Maine (September 2011, Latitude: 44°37’N) and Panama City, Panama (January 2012, Latitude: 8°58’N), placed individually on vials and then shipped to the laboratory where they were maintained as isofemale lines. Isofemale lines were maintained at 25°C on a standard yeast-cornmeal-agar food medium. Experimental animals were generated from these lines in Spring 2012. To generate experimental animals for each line, 40 freshly laid eggs were picked and placed onto a vial, which was then incubated at either of two temperature treatments, 21°C or 29°C, at synchronized 12:12 hour Light:Dark. Virgin males were collected within 3 hours after eclosion. After 48 hours an individual male was randomly picked from each isofemale line without anesthetics to create a biological replicate for each population and temperature; a total of three biological replicates were created for each population/temperature combination. Flies were frozen in Trizol and stored at -80°C before RNA was extracted following a standard Trizol-chloroform extraction protocol. The numbers of isofemale lines contributing to each biological replicate were as follows. For the 21°C treatment, we used 29 *D*. *melanogaster* Panama strains and 30 Maine strains; we used 17 *D*. *simulans* Panama strains and 29 Maine strains. For the 29°C treatment, we used 30 *D*. *melanogaster* Panama strains and 30 Maine strains; we used 15 *D*. *simulans* Panama strains and 30 Maine strains. We used a relatively large number of strains from each population with three replicates. Thus, although we have no method of partitioning expression variance across individuals within and between populations, we make the reasonable assumption that for the majority of genes we obtain useful estimates of mean population expression phenotypes.

### Sequencing, Assembly and Data Filtering

Poly(A)+ RNA was prepared from an aliquot of each total RNA sample. Individual libraries were constructed using the Illumina Truseq RNA kit with insert size 170–200 bp and sequenced on an Illumina HiSeq machine using paired-ends chemistry and 90 cycles. Clean reads were deposited to NCBI under BioProject number PRJNA260940 and SRA number SRP047141. Filtered, clean reads for each sample/replicate were aligned independently to a reference genome using the Bowtie-based TopHat program [74]. *D*. *melanogaster* reads were mapped to reference genome (FlyBase r5.55). *D*. *simulans* reads were mapped to dsimv2 [75] and reference genome (FlyBase r1.4). Only uniquely mapped reads (Q >20 for bases and Q >30 for reads) were kept for further analysis. For each species, there were more than 300 million paired-end reads (60 gigabases) aligned to the each genome.

### Gene Differential Expression Analysis

We measured differential expression with DESeq2 [76] (version 1.4.5), edgeR [77] (version 3.0.8) and voom-limma [78] (version 3.20.8). We first adopted Bedtools to estimate a read count for each gene. Genes with a minimum 10 counts average were kept for further analysis. We then used the Bioconductor package (version 2.14) in R to analyze read counts. We used DESeq2, edgeR, and voom-limma with empirical Bayes estimation and exact tests based on a negative binomial model [77] or linear modeling [78]. The Benjamini—Hochberg procedure was used to control the false discovery rate [79] for all methods. We observed that the different methods returned highly consistent count estimates. Here were present results from DESeq2 differentially expressed genes because these results showed the greatest consistency compared to the other two methods. For gene plasticity results, only genes showing significant differences between temperatures by at least two methods were kept for further analysis. All major conclusions related to gene lists, GO enrichment, and parallel expression differences between species were supported by either method of normalization. We also used Cufflinks [80] to calculate the expression value (FPKM) with upper quantile normalization. To examine differential expression of genes/transcript between different samples, we used Cuffdiff2 [80] with all three biological replicates, and then obtained genes/transcripts with significant differences (P <0.05 and the FDR <0.05 after Benjamini-Hochberg correction for multiple-testing). Using this program, we also estimated isoform usage for each gene.

### Orthologous Genes Analysis

To infer orthologous genes in *D*. *melanogaster* and *D*. *simulans*, we used MCscan [81] to determine synteny relationships for all genes in both reference genomes. We then identified reciprocal best hits for all genes in each pairwise species comparison using BLASTP (at alignment length >50%, similarity >70%). Genes having reciprocal best hits and shared synteny were defined as orthologous. Single-copy genes that were reciprocal best hits but were not syntenic were also defined as orthologs because such cases are easily explained by genomic rearrangements. Using this approach we identified about 11,000 genes as orthologous in the two species, of which, 10,085 genes were expressed in the dataset, and thus used to investigate parallel gene expression differentiation. We used modENCODE developmental stage RNAseq data from whole male and whole female adults [44] to identify male-biased genes in *D*. *melanogaster*. We used modENCODE *D*. *simulans* male (NCBI SRA SRR166817 and SRR166818) and female (NCBI SRA SRR166815 and SRR166816) to identify *D*. *simulans* male-biased genes. Only high quality unique reads were mapped to each reference sequence using TopHat, which were then used to estimate expression with Cufflinks/Cuffdiff2. For genes that showed FPKM >2 in the male sample, if expression was more than two fold greater than that observed for the female sample, we defined the gene as male-biased. Using this method, we identified 3920 and 3546 such genes for *D*. *melanogaster* and *D*. *simulans*, respectively. We calculated the tissue specificity index (tau) of each gene using FlyAtlas data [42]. For each gene, we obtain a tau value and recorded the tissue in which expression was highest. To identify testis-biased genes we used FlyAtlas data with tau >0.9 [41]. We also downloaded male-specific genes from Graveley et al. [44].

### Data Comparison and Analysis

We used a FDR adjusted P-value <0.05 as the cutoff for differential expression. For geographic comparisons we also calculated absolute fold changes for all genes to estimate genome-wide expression variation. The fold changes were transformed to absolute fold change values (Panama population expression as 1). Differential gene expression enrichment was tested using hypergeometric or *χ* ^*2*^ tests. All gene location, UTR, CDS information used in the analysis were downloaded and extracted from FlyBase r5.55. GEI interaction was estimated following Levine et al. [51] using the limma [78] package. Inversion breakpoints were from Corbett-Detig and Hartl [82]. We generated empirical distributions to determine whether differentially expressed genes are physically clustered. We randomly picked a number genes on each chromosome arm corresponding to the observed differentially expressed genes, calculated the distance between genes, and repeated this 1000 times. We compared the median distances separating genes in the observed differentially expressed genes and the randomly sampled genes.

### Splicing Isoforms

We extracted the reads that spanned the junctions from TopHat-mapped bam files. For each such read we identified the junction site (including intron location, chromosome location and left and right of the junction sites) and calculated the read count (coverage) for each junction (as well as introns). We then extracted annotated intron information from FlyBase (data extracted from *D*. *melanoagaster* FlyBase r5.55 and *D*. *simulans* r1.4) and compared the introns/junctions between identified ones with annotated introns. We then fed the junction counts into R and calculated if splice junction abundance was heterogeneous with FDR <0.05. For alternative splicing, we used Cufflinks and Cuffdiff2 to detect different splicing isoforms and their expression with FDR <0.05. To identify shared differential junction use between species, we converted *D*. *simulans* junction/intron coordinates to the *D*. *melanogaster* assembly using UCSC liftOver.

### Functional Annotation

We used DAVID [33] to compare enrichment for functional terms among groups of genes. DAVID’s tools use a modified Fisher’s exact test (the EASE score) to determine the extent of enrichment for a subset of genes compared to a specified background. GO categories that were significantly enriched at a false discovery rate <0.05 and Bonferroni corrected P value <0.05 were used. We also used GOTermFinder [34] to confirm the DAVID results for *D*. *melanogaster* genes.

### Transcriptomic *F*_*ST*_ Analysis

We took advantage of the high coverage RNA-seq data to calculate transcriptomic *F*_*ST*_ in both *D*. *melanogaster* and *D*. *simulans*. For reads that aligned to *D*. *melanogaster* and *D*. *simulans* genome, we removed sites that had coverage <10 and those for which a SNP was supported by only a single read. SNPs and coverage were then calculated and extracted by SAMtools mpileup [83]. SNP frequencies for each population (3 biological replicates pooled together; median coverage for the SNPs were 494 for *D*. *melanogaster* and 220 for *D*. *simulans*) were then calculated. In order to obtain the first dataset of population SNP differentiation, we calculated a non-conventional *F*_*ST*_ using RNA-seq data. SNP *F*_*ST*_ were estimated by Popoolation2 [84]; we removed SNPs with frequency <0.01. We then identified SNPs in top 1%, 0.5% and 0.25% *F*_*ST*_ tail on each chromosome arm. Each SNP was assigned to one of three categories, 5’ UTR, 3’ UTR or CDS. We then determined the overlap between genes associated with tail SNPs and those showing geographic differential expression at 29°C or 21°C. For *D*. *simulans*, we used FlyBase r1.4 CDS coordinates. For 3’ UTR and 5’ UTR, we used the UCSC liftOver (https://genome.ucsc.edu/cgi-bin/hgLiftOver) coordinates from *D*. *melanogaster* UTRs. We determined whether differentiated expressed genes were enriched in locations with high *F*_*ST*_ using 1kb *F*_*ST*_ data generated by Reinhardt et al. [15]. We found the conclusions using transcriptomic *F*_*ST*_ were consistent with conclusions using genomic *F*_*ST*_ from Reinhardt et al. [15]. Hypergeometric tests were used to calculate the P-values and expressed UTRs, CDSs or mRNAs were used as the population size for each test.

To determine whether the number of shared genes/CDS with *F*_*ST*_ outliers in *D*. *simulans* and *D*. *melanogaster* is influenced by gene/CDS size, we carried out 1000 independent bootstraps to obtain an empirical distribution of shared outlier genes considering the number of SNPs in each gene. We first counted the number of correlated outlier SNPs in the genes that have 0.25% *F*_*ST*_ tail, for example in *D*. *melanogaster* there were 386 genes having one SNP outlier, 196 genes having two SNP outliers, 2 to 86 genes that has 3 to 9 SNP outliers and 6 genes with more than 10 SNP outliers. In *D*. *simulans* we calculated the same SNP outlier properties for each gene. We then randomly picked genes/CDS that have number SNPs equal to or greater than the observed SNPs in the shared genes in each species, and then calculated the number of shared orthologous genes/CDS in *D*. *melanogaster* and *D*. *simulans*. After repeating 1000 times, we obtained the empirical distribution and P-values.

## Supporting Information

S1 Fig Plot for correlation of the expression between biological replicates. (TIFF)

S2 Fig Histogram of geographic expression fold changes in *D*. *melanogaster* and *D*. *simulans*. A) Fold changes (log2) for Panama vs. Maine population at 21°C and 29°C. B) Fold changes (log2) for differentially expressed genes in Panama vs. Maine population at 21°C and 29°C.
(TIFF)

S3 Fig Histogram of temperature plasticity fold changes in *D*. *melanogaster* and *D*. *simulans*. A) Fold changes (log2) for 21°C vs. 29°C in each population. B) Fold changes (log2) for genes showing differential expression at 21°C vs. 29°C in each population.
(TIFF)

S1 Text Supplementary text for gene expression comparison and expression fold correlation. (DOCX)

S1 Table Samples and total sequencing read number. (DOCX)

S2 Table List of differentially expressed genes for low vs. high latitude in *D*. *melanogaster* and *D*. *simulans*. A) *D*. *melanogaster* Panama vs. Maine at 29°C, B) *D*. *melanogaster* Panama vs. Maine at 21°C, C) *D*. *simulans* Panama vs. Maine at 29°C and D) *D*. *simulans* Panama vs. Maine at 21°C.
(XLSX)

S3 Table GO enrichment of geographically differentially expressed genes in *D*. *melanogaster* and *D*. *simulans*. (XLSX)

S4 Table Top 50 most geographically differentially expressed genes of *D*. *melanogaster* at 29°C and 21°C. (XLSX)

S5 Table List of differentially expressed transcription factors for low vs. high latitude in *D*. *melanogaster* and *D*. *simulans*. A) *D*. *melanogaster* Panama vs. Maine at 29°C, B) *D*. *melanogaster* Panama vs. Maine at 21°C, C) *D*. *simulans* Panama vs. Maine at 29°C and D) *D*. *simulans* Panama vs. Maine at 21°C.
(XLSX)

S6 Table Geographic differential expression for testis-biased and male-specific genes. (DOCX)

S7 Table Panama vs. Maine differential expression for genes in regions spanned by cosmopolitan inversions. (DOCX)

S8 Table Parallel differentially expressed genes in *D*. *melanogaster* and *D*. *simulans*. A) At 29°C and B) at 21°C. Columns A and N are orthologous genes between *D*. *melanogaster* and *D*. *simulans*.
(XLSX)

S9 Table List of genes showing temperature plasticity in *D*. *melanogaster* and *D*. *simulans*. A) *D*. *melanogaster* Panama 21°C vs. 29°C, B) *D*. *melanogaster* Maine 21°C vs. 29°C, C) *D*. *simulans* Panama 21°C vs. 29°C and D) *D*. *simulans* Maine 21°C vs. 29°C.
(XLSX)

S10 Table Shared temperature plasticity genes in *D*. *melanogaster* and *D*. *simulans*. A) Maine populations B) Panama populations.
(XLSX)

S11 Table Shared temperature plasticity genes for Australia and the Americas. A) High latitude, Tasmania and Maine B) Low latitude, Queensland and Panama.
(XLSX)

S12 Table Outlier SNPs in differentially expressed genes and enrichment P-values. (DOCX)

S13 Table Genes that have 0.5% and 1% tail *F*_*ST*_ outlier in both 5' and 3' UTR. (XLSX)

S14 Table List of introns/splicing junctions showing differential low vs. high latitude usage in *D*. *melanogaster* and *D*. *simulans*. A) *D*. *melanogaster* Panama vs. Maine at 29°C, B) *D*. *melanogaster* Panama vs. Maine at 21°C, C) *D*. *simulans* Panama vs. Maine at 29°C and D) *D*. *simulans* Panama vs. Maine at 21°C.
(XLSX)

S15 Table Shared geographically differentiated splice junction use for *D*. *melanogaster* and *D*. *simulans*. (XLSX)

S16 Table Significant GEI genes in *D*. *melanogaster* and *D*. *simulans*. (XLSX)

S17 Table GO enrichment of significant GEI genes in *D*. *melanogaster*. (XLSX)
