# Supplementary figures and images for: Parallel Gene Expression Differences between Low and High Latitude Populations of Drosophila melanogaster and D. simulans
Source: PLoS Genet. 2015 May 7;11(5):e1005184. doi: 10.1371/journal.pgen.1005184 (PMC4423912; doi:10.1371/journal.pgen.1005184)

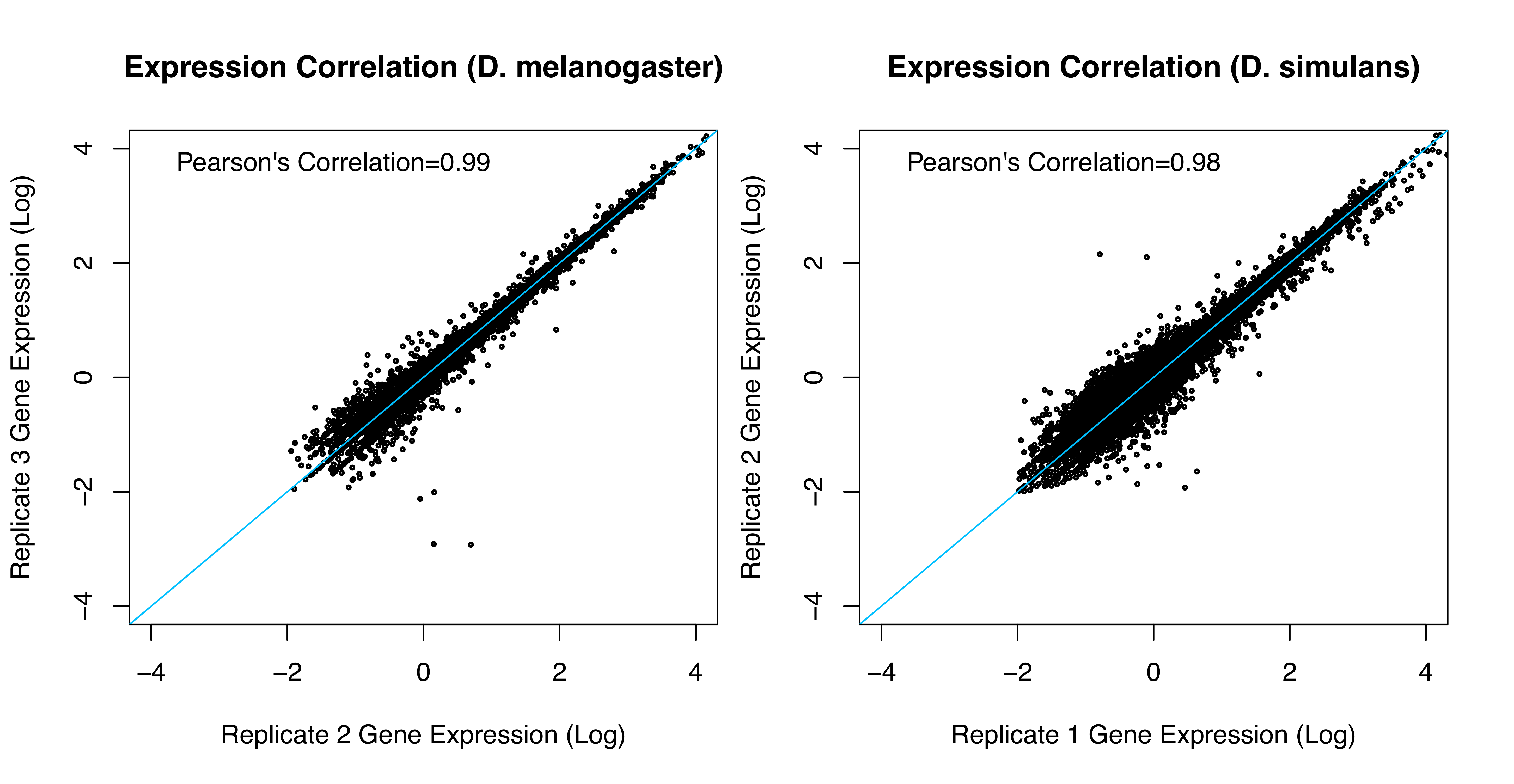

Supplement: S1 Fig — (TIFF) [file pgen.1005184.s001.tiff]

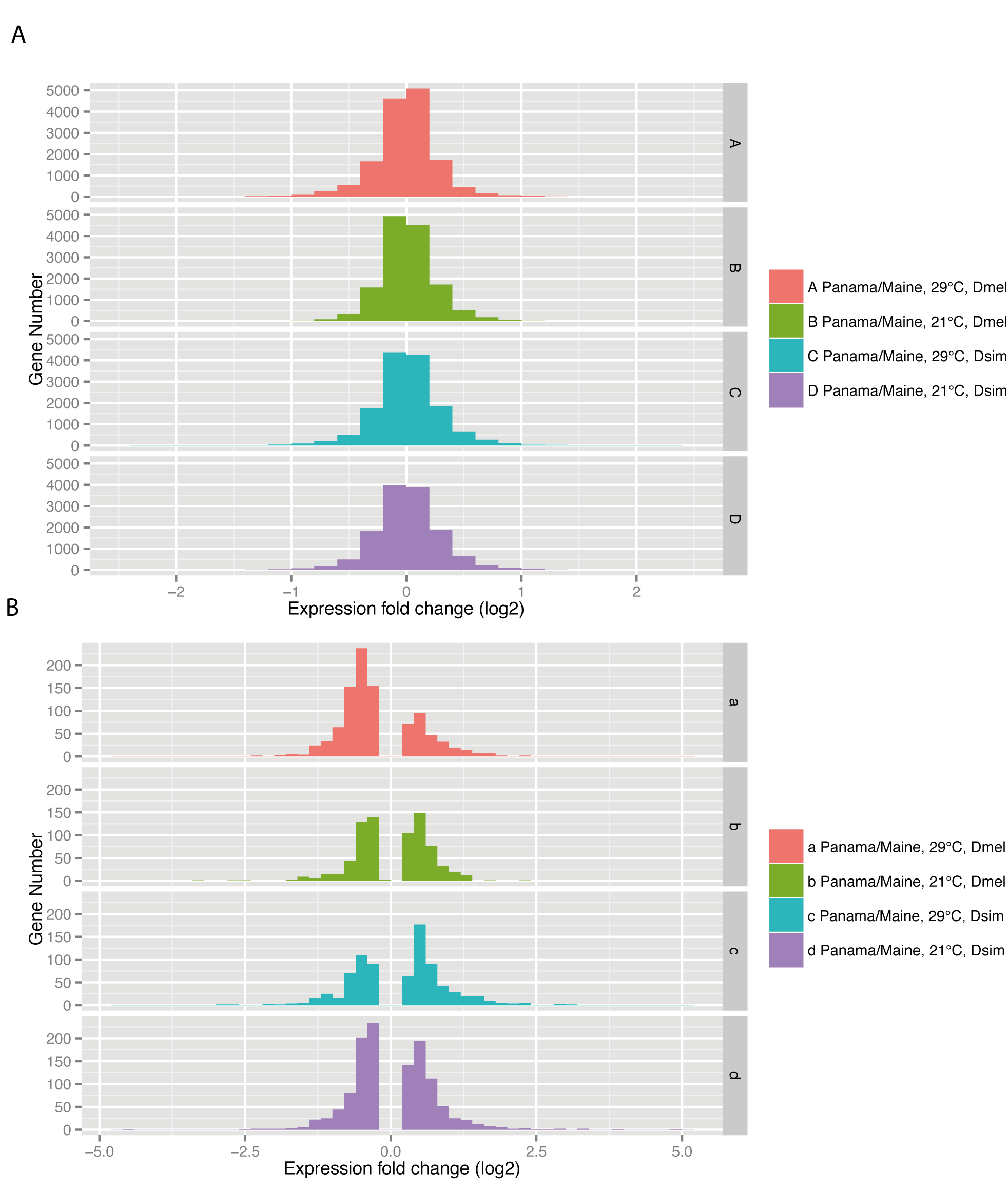

Supplement: S2 Fig — A) Fold changes (log2) for Panama vs. Maine population at 21°C and 29°C. B) Fold changes (log2) for differentially expressed genes in Panama vs. Maine population at 21°C and 29°C. (TIFF) [file pgen.1005184.s002.tiff]

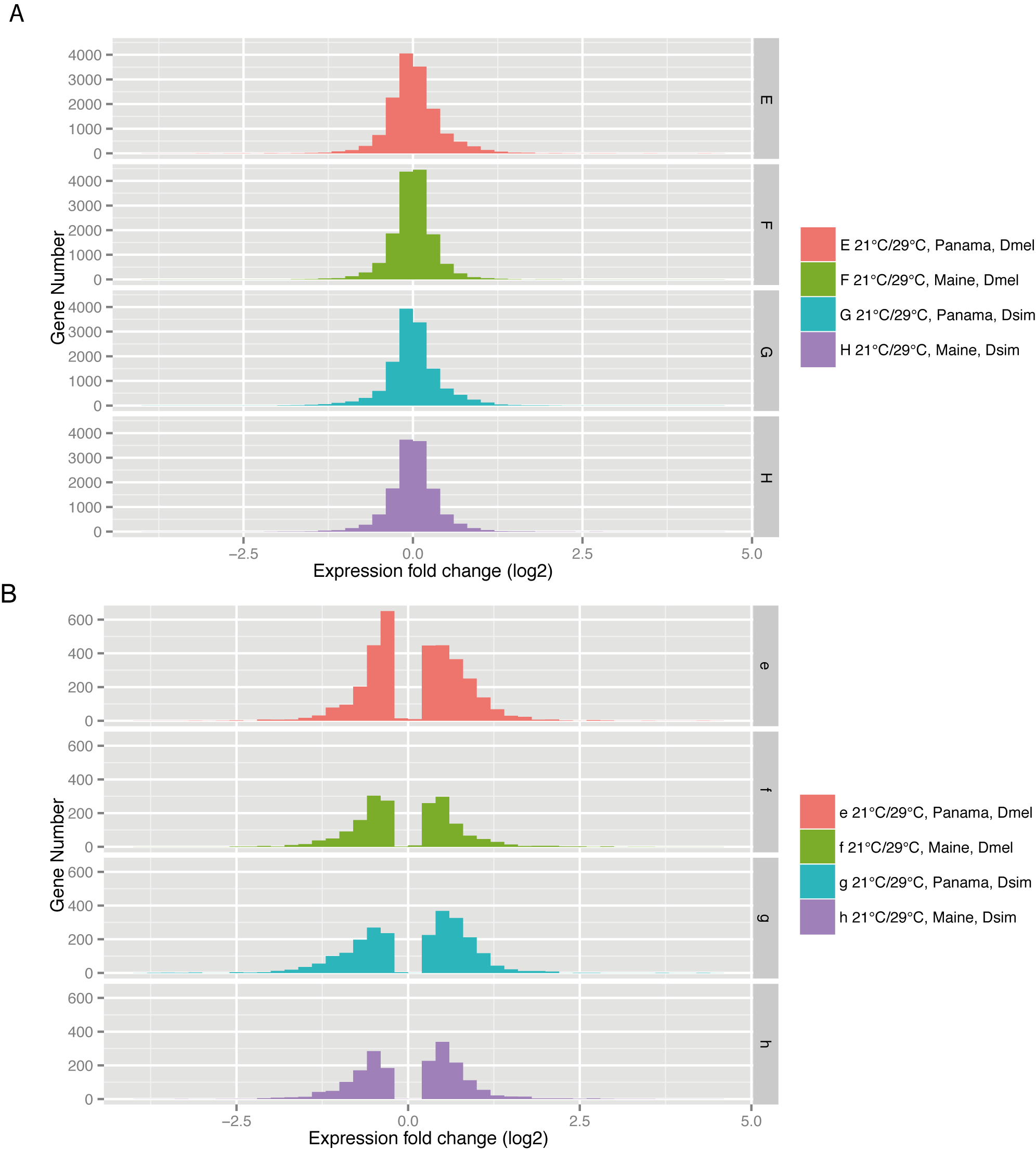

Supplement: S3 Fig — A) Fold changes (log2) for 21°C vs. 29°C in each population. B) Fold changes (log2) for genes showing differential expression at 21°C vs. 29°C in each population. (TIFF) [file pgen.1005184.s003.tiff]
