# Supplementary material for: Parallel Gene Expression Differences between Low and High Latitude Populations of Drosophila melanogaster and D. simulans
Source: PLoS Genet. 2015 May 7;11(5):e1005184. doi: 10.1371/journal.pgen.1005184 (PMC4423912; doi:10.1371/journal.pgen.1005184)
Supplement: S1 Text — (DOCX) [file pgen.1005184.s004.docx]

S1 Text for “Parallel Gene Expression Differences Between Low and High Latitude Populations of *Drosophila melanogaster* and *D. simulans* ”

#### Comparison to previous transcriptome data

#### We compared our list of geographically differentially expressed genes with that of Huylmans and Parsch 2014 [37], which presented a transcriptome analysis of Malpighian tubules samples from Africa and the Netherlands. They reported 484 genes that showed population expression differences of > 20% in male flies. We observed 30 and 42 genes (a total of 63 genes) in common with their list, including *Cyp6g1*. However, direct comparison of these experiments is compromised by differences in source populations, tissues, and rearing temperatures.

#### We compared our list to Chen et al. [39], a tilling array analysis using East Coast Australia larvae samples which were reared at 25°C. Compare with the 28 genes (table 1 in [39]) that showed significant differential expression between south and north, we found 10 genes were significantly differentially expressed at least for one temperature in our treatment, with 6 in the same direction. We also found the *srp* gene, the main case Chen et al. [39] studied, showed the same expression pattern at 21°C in North America dataset that temperate population showing higher expression.

#### Expression fold correlation between two species

#### To investigate whether parallelism extends to the magnitude of population differences in transcript abundance we estimated the interspecific correlation (in fold change) for genes exhibiting parallel significantly different expression. We observed a remarkably strong correlation (Pearson’s R^2^ of log2 fold changes = 0.80). As this correlation was calculated using a biased subset of the data (genes showing parallel expression differences), making it difficult to interpret the correlation. Thus, we estimated the correlation of geographic fold-change differences for the two species using all differentially expressed genes. The Pearson’s R^2^ was 0.53 for genes showing the same direction of expression, compared to R^2^ of 0.34 for genes showing opposite direction of expression differences for the two species. We then calculated the geographic fold change difference for all expressed genes in the two species (regardless of whether expression differences were significant. For this dataset the Pearson’s R^2^ was 0.37 for genes with parallel expression differences and 0.26 for opposite expression differences. The stronger correlation in fold change for same vs. opposite direction differences is consistent with the idea that parallel selection responses play a role in parallel expression differences in these two species.
