# Supplementary material for: Parallel Gene Expression Differences between Low and High Latitude Populations of Drosophila melanogaster and D. simulans
Source: PLoS Genet. 2015 May 7;11(5):e1005184. doi: 10.1371/journal.pgen.1005184 (PMC4423912; doi:10.1371/journal.pgen.1005184)
Supplement: S1 Table — (DOCX) [file pgen.1005184.s005.docx]

S1 Table. Samples and total sequencing read number.

| Species | Population | Rear Temperature | Replicate1  (Million) | Replicate2  (Million) | Replicate3  (Million) | Total Reads  (Million) |
| --- | --- | --- | --- | --- | --- | --- |
| *D. melanogaster* | Panama | 21°C | 31.6 | 28.6 | 28.3 | 88.5 |
| *D. melanogaster* | Panama | 29°C | 32.8 | 27.1 | 26.0 | 85.9 |
| *D. melanogaster* | Maine | 21°C | 29.9 | 22.9 | 31.1 | 83.9 |
| *D. melanogaster* | Maine | 29°C | 29.4 | 28.7 | 31.3 | 89.4 |
| *D. simulans* | Panama | 21°C | 30.8 | 31.6 | 29.5 | 91.9 |
| *D. simulans* | Panama | 29°C | 34.0 | 37.8 | 31.5 | 103.3 |
| *D. simulans* | Maine | 21°C | 29.1 | 26.2 | 25.4 | 80.7 |
| *D. simulans* | Maine | 29°C | 28.0 | 26.2 | 29.3 | 83.5 |

The numbers presented are million paired ends number. The total sequencing data needs to multiply 2×90bp.
