# Supplementary material for: Parallel Gene Expression Differences between Low and High Latitude Populations of Drosophila melanogaster and D. simulans
Source: PLoS Genet. 2015 May 7;11(5):e1005184. doi: 10.1371/journal.pgen.1005184 (PMC4423912; doi:10.1371/journal.pgen.1005184)
Supplement: S6 Table — (DOCX) [file pgen.1005184.s010.docx]

S6 Table. Geographic differential expression for testis-biased and male-specific genes

| Species | Genotype | Differentially expressed genes | Testis-biased genes | P-value | Male- specific genes | P-value |
| --- | --- | --- | --- | --- | --- | --- |
| *D. melanogaster* | Panama vs. Maine at 29°C | 980 | 43 | 1 | 17 | 0.9999 |
|  | Panama vs. Maine at 21°C | 759 | 47 | 0.9999 | 11 | 0.9999 |

Total expressed genes were 14006 in *D. melanogaste*. Testis-biased genes were generated using FlyAtlas data with a tau>0.9, and the total number were 1492. Male-specific genes were from Graveley et al. 2011 (add ref), 560 of 572 genes were expressed in our dataset. *P-value* were calculated by hypergeometric test about the possibility that if there is over-representation of testis or male specific genes in the differentially expressed genes.
