# Supplementary material for: Parallel Gene Expression Differences between Low and High Latitude Populations of Drosophila melanogaster and D. simulans
Source: PLoS Genet. 2015 May 7;11(5):e1005184. doi: 10.1371/journal.pgen.1005184 (PMC4423912; doi:10.1371/journal.pgen.1005184)
Supplement: S7 Table — (DOCX) [file pgen.1005184.s011.docx]

S7 Table. Panama vs. Maine differential expression for genes in regions spanned by cosmopolitan inversions.

| Inversion | Temperature | Distal breakpoint  (M bp) | Proximal breakpoint  (M bp) | Gene number | Differentially expressed genes | *P-*value |
| --- | --- | --- | --- | --- | --- | --- |
| *In(2L)t* | 29°C | 2.226 | 13.154 | 1463 | 92 | 0.906 |
| *In(2L)t* | 21°C | 2.226 | 13.154 | 1463 | 72 | 0.855 |
| *In(2R)NS* | 29°C | 11.279 | 16.164 | 669 | 34 | 0.987 |
| *In(2R)NS* | 21°C | 11.279 | 16.164 | 669 | 33 | 0.749 |
| *In(3L)P* | 29°C | 3.173 | 16.302 | 1430 | 110 | 0.122 |
| *In(3L)P* | 21°C | 3.173 | 16.302 | 1430 | 78 | 0.491 |
| *In(3R)K* | 29°C | 7.576 | 21.966 | 1815 | 162 | 4.78E-05 |
| *In(3R)K* | 21°C | 7.576 | 21.966 | 1815 | 123 | 1.01E-03 |
| *In(3R)Mo* | 29°C | 17.233 | 24.857 | 993 | 71 | 0.435 |
| *In(3R)Mo* | 21°C | 17.233 | 24.857 | 993 | 61 | 0.145 |
| *In(3R)P* | 29°C | 12.258 | 20.57 | 998 | 111 | 3.89E-08 |
| *In(3R)P* | 21°C | 12.258 | 20.57 | 998 | 81 | 3.66E-05 |

The locations of inversions were extracted from Corbett-Detig and Hartl (82). Gene number refers to expressed genes in regions spanned by inversion. *P*-values were from hypergeometric test.
