# Supplementary material for: Parallel Gene Expression Differences between Low and High Latitude Populations of Drosophila melanogaster and D. simulans
Source: PLoS Genet. 2015 May 7;11(5):e1005184. doi: 10.1371/journal.pgen.1005184 (PMC4423912; doi:10.1371/journal.pgen.1005184)
Supplement: S12 Table — (DOCX) [file pgen.1005184.s016.docx]

S12 Table. Outlier SNPs in differentially expressed genes and enrichment p-values.

| **Species** | **Temperature** | **Gene annotation** | **SNP Fst tail** | **Number differentially expressed genes with tail SNP for different annotations** | **Number differentially expressed genes** | **Enrichment P-value** |
| --- | --- | --- | --- | --- | --- | --- |
| *D. melanogaster* | 21°C | 3' UTR | SNP Fst 1% tail | 65 | 531 | 0.016 |
|  |  | 5' UTR | SNP Fst 1% tail | 44 | 471 | 0.011 |
|  |  | CDS | SNP Fst 1% tail | 138 | 634 | 0.065 |
|  | 29°C | 3' UTR | SNP Fst 1% tail | 116 | 611 | 2.83E-10 |
|  |  | 5' UTR | SNP Fst 1% tail | 51 | 623 | 0.062 |
|  |  | CDS | SNP Fst 1% tail | 239 | 1091 | 0.000 |
| *D. simulans* | 21°C | 3' UTR | SNP Fst 0.25% tail | 17 | 531 | 0.259 |
|  |  | 5' UTR | SNP Fst 0.25% tail | 17 | 471 | 0.004 |
|  |  | CDS | SNP Fst 0.25% tail | 55 | 634 | 0.003 |
|  | 29°C | 3' UTR | SNP Fst 0.25% tail | 47 | 531 | 2.13E-13 |
|  |  | 5' UTR | SNP Fst 0.25% tail | 17 | 471 | 0.004 |
|  |  | CDS | SNP Fst 0.25% tail | 66 | 634 | 4.25E-06 |
| *D. simulans* | 21°C | 3' UTR | SNP Fst 0.25% tail | 11 | 611 | 0.999 |
|  |  | 5' UTR | SNP Fst 0.25% tail | 15 | 623 | 0.555 |
|  |  | CDS | SNP Fst 0.25% tail | 88 | 1091 | 0.001 |
|  | 29°C | 3' UTR | SNP Fst 0.25% tail | 17 | 531 | 0.259 |
|  |  | 5' UTR | SNP Fst 0.25% tail | 11 | 471 | 0.219 |
|  |  | CDS | SNP Fst 0.25% tail | 51 | 634 | 0.015 |
| D. melanogaster | 21°C | Gene | 1kb Fst 1% tail | 42 | 759 | 0.001 |
|  |  | Gene | 1kb Fst 2.5% tail | 66 | 759 | 0.140 |
|  |  | Gene | 1kb Fst 5% tail | 110 | 759 | 0.116 |
|  | 29°C | Gene | 1kb Fst 1% tail | 62 | 980 | 3.58E-07 |
|  |  | Gene | 1kb Fst 2.5% tail | 119 | 980 | 1.71E-08 |
|  |  | Gene | 1kb Fst 5% tail | 181 | 980 | 4.57E-08 |

The comparisons for differential expression were Panama vs. Maine populations. “Number differentially expressed genes” refers to the number of differentially expressed genes that have annotated UTRs or CDS. P-values were from hypergeometric test.
